# Imitation of snack food intake among normal-weight and overweight children

**DOI:** 10.3389/fpsyg.2013.00949

**Published:** 2013-12-18

**Authors:** Kirsten E. Bevelander, Anna Lichtwarck-Aschoff, Doeschka J. Anschütz, Roel C. J. Hermans, Rutger C. M. E. Engels

**Affiliations:** Developmental Psychopathology Department, Behavioural Science Institute, Radboud University NijmegenNijmegen, Netherlands

**Keywords:** imitation, mimicry, social modeling, children, overweight, eating behavior

## Abstract

This study investigated whether social modeling of palatable food intake might partially be explained by the direct imitation of a peer reaching for snack food and further, assessed the role of the children's own weight status on their likelihood of imitation during the social interaction. Real-time observations during a 10-min play situation in which 68 participants (27.9% overweight) interacted with normal-weight confederates (instructed peers) were conducted. Children's imitated and non-imitated responses to the confederate's food picking movements were compared using a paired sample *t-*test. In addition, the pattern of likelihood of imitation was tested using multilevel proportional hazard models in a survival analysis framework. Children were more likely to eat after observing a peer reaching for snack food than without such a cue [*t*_(67)_ = 5.69, *P* < 0.0001]. Moreover, findings suggest that children may display different imitation responses during a social interaction based on their weight status (*HR* = 2.6, *P* = 0.03, 95% *CI* = 1.09–6.20). Overweight children were almost twice as likely to imitate, whereas normal-weight children had a smaller chance to imitate at the end of the interaction. Further, the mean difference in the likelihood of imitation suggest that overweight children might be less likely to imitate in the beginning of the interaction than normal-weight children. The findings provide preliminary evidence that children's imitation food picking movements may partly contribute to social modeling effects on palatable food intake. That is, a peer reaching for food is likely to trigger children's snack intake. However, the influence of others on food intake is a complex process that might be explained by different theoretical perspectives.

## Introduction

Within the past 30 years, the prevalence of obesity has more than doubled in children and tripled in adolescents in the United States (CDC, 2012). As people often eat in the presence of others, the social environment is an important research area. Social modeling literature has provided ample evidence that the presence of others affects people's food intake. Numerous studies have shown that an individual's food intake is affected by their eating companion's intake by means of an experimental design in which naïve participants are exposed to experimental confederates who are instructed to eat different amounts of food (Herman et al., 2003; Herman and Polivy, 2005; Hermans et al., 2009a; Bevelander et al., 2012). Social modeling studies have shown that people are likely to eat more or less food when their eating companion eats more or less. Notably, modeling of food intake has been consistently found among adults, adolescents and children regardless of one's hunger or satiety levels and in different eating contexts (Goldman et al., 1991; Herman and Polivy, 2005; Hermans et al., 2009b; McFerran et al., 2010; Feeney et al., 2011).

A potential explanation for social modeling effects in food intake is that people are believed to monitor other's food intake in order to resolve their own uncertainty about the appropriate amount to consume (Herman et al., 2003). Therefore, they use the food intake of others as a guideline or norm for their own amount of intake. Besides people's general need for an informational guideline, it is suggested that people have social motives to conform to others eating because conformity can lead to social acceptance or approval (Herman et al., 2003; Vartanian et al., 2007; Hermans et al., 2008, 2009a; Bevelander et al., 2012). For example, a study on young female adolescents showed that the ones who were primed with feelings of social acceptance followed the food intake of their peers less often than those who were not primed with social acceptance (Robinson et al., 2011). This line of reasoning overlaps with literature on behavioral imitation proposing that people imitate the behavior (e.g., imitation of gestures, facial expression, etc.) of others as a way to affiliate (Lakin and Chartrand, 2003). This imitation process is assumed to occur unconsciously by a tight neural link between perception and action (Chartrand and Bargh, 1999; Chartrand et al., 2009). That is, perceiving another person's movements may trigger and activate one's own motor system for the same movement (Lakin and Chartrand, 2003; Iacoboni, 2009). A new line of research investigates whether a person's consumption behavior is directly imitated or copied by another person. For example, studies on alcohol consumption have shown that young adults might imitate the drinking behavior of peers and movie actors by taking a sip directly after the observed person had taken a sip (Larsen et al., 2010; Koordeman et al., 2011). In the domain of eating, seeing another person taking a bite or snack might trigger a similar response. One study has demonstrated that female young adults imitated the bites of their eating companion (Hermans et al., 2012). That is, young women were more likely to take a bite of their meal directly after their eating companion had taken a bite. Based on the findings of these studies in young adults, the aim of the current study was to investigate whether the movement of a peer reaching for food (“food picking movements”) would trigger a similar response in young boys and girls as well.

Although people generally tend to use other's food intake as a guideline for their own intake, it is suggested that the extent to which they model other's food intake depends on a variety of personal and situational characteristics. The scarce studies on imitation have shown that individuals are more likely to imitate a peer at the beginning than at the end of a social interaction (Koordeman et al., 2011; Hermans et al., 2012). A tentative explanation for this finding is that affiliation goals affect behavioral imitation processes. Social psychology research suggests that people imitate each other's behavior in order to affiliate with others and to create social bonding (Baumeister and Leary, 1995; Lakin et al., 2003). One could argue that affiliation or ingratiation strategies are stronger at the beginning than at the end of a social interaction because people get to know each other during that time. Therefore, the occurrence of imitation might decrease over the duration of a social interaction when people feel more at ease (Hermans et al., 2012).

In social modeling literature, there is evidence to suggest that children's weight status might have a differential impact on the degree to which children model their peer's food intake (Salvy et al., 2007; Bevelander et al., 2012). Therefore, the pattern of imitation during a social interaction might be relevant to examine in this context. Given that there is a strong association between overweight status and stigmatization (Herman et al., 2003; Puhl and Latner, 2007), overweight children might be more inclined to affiliate with peers by imitating their food intake compared to normal-weight children during a social interaction. In contrast, normal-weight children might imitate less because they feel more socially accepted in general and have a higher social confidence level than overweight children (Pierce and Wardle, 1997; Strauss and Pollack, 2003). To the authors knowledge, no previous studies have investigated how these imitation processes unfold in real-time among normal-weight and overweight children.

Given that this is the first study on imitation of palatable food intake among children, the current study is exploratory in nature and aims to broaden the existing scope of knowledge by investigating whether children imitate the food picking movements of a peer. It was hypothesized that children would directly imitate the food picking movements of a same-sex peer. In addition, in line with previous research among young adults, it was explored whether the likelihood of imitation changed during the social interaction and whether this differed between normal-weight and overweight children.

## Experimental methods

### Participants and procedure

This sample was taken from a larger study investigating social modeling behavior of food intake among normal-weight and overweight children at Dutch primary schools. Each participant was placed opposite a same-sex normal-weight confederate (instructed peer) at a table with a 100-piece puzzle, two bowls of test food (chocolate-coated peanuts), and two glasses of water. The confederates were instructed to pick one chocolate-coated peanut when covertly signaled by the experimenter (i.e., 10 signals, one every minute during the 10-min social interaction). The experimenter signaled the confederates with a small vibrating device (a buzzer) which they wore in either their pocket or sock. The experimenter set the buzzer interval to control for the timing of food intake. Each session was recorded digitally. A detailed description of the design and methodology of the original study can be found elsewhere (Bevelander et al., 2012). As this study investigated the imitation of food picking movements (i.e., eating cues), the present study included participants (*n* = 72) who were in the “high intake” condition only. Four dyads were excluded from analysis because the video records were not suitable for coding (*n* = 3) or questionnaire information was incomplete (*n* = 1). The final sample consisted of 68 children (50% boys; 27.9% overweight) with a mean age of 8.56 (±1.46) years.

### Measures

#### Food picking

An apparent cue in the onset for food intake was the moment after the children had moved their arm and hand toward the food bowl and picked a chocolate-covered peanut (i.e., the test food). So, the exact time in seconds at which the confederates' and the participants' fingers touched and picked a chocolate peanut from the bowl was coded from the video recordings. Similar to a previous study on food intake, a 5-s time window was used to test whether imitation could occur (Hermans et al., 2012). The 5-s time frame started on the moment that the confederate's fingers picked a chocolate peanut.

To investigate the participant's imitation of the confederate's food picking, a distinction was made between “imitated picking” and “non-imitated picking.” The participant picking a chocolate peanut within 5s after the confederate had picked a chocolate-coated peanut, was defined as imitated picking and coded as “1” (i.e., those instances were considered events in the second analyses by means of a multi-level Cox regression model). A participant taking a chocolate peanut after the 5-s interval was defined as non-imitated picking and coded as “0.” When participants picked more than one chocolate peanut at the same time, this was still regarded as a single food pick because they had reached for the food bowl and picked food once. As some of the confederates missed one of the 10 covertly given signals (*n* = 5) to pick test food, a total of *n* = 675 food picking occasions were used from the final sample of 68 children. Data were coded by two trained coders who coded the data independently and were blind to the time frame of imitation.

#### Total number of times participant picked test food

The occurrence of imitation is proportionate to the total number of times participants picked a chocolate peanut. That is, if a participant picks a lot of chocolate peanuts, the occurrence of imitation might be higher due to chance alone (picking a peanut might fall into the 5-s interval due to the high rate of participants' picking and not because of imitation). Therefore, the total number of times that participants picked chocolate-covered peanuts was controlled for.

#### Body weight

Participants' height and weight were measured objectively by the experimenter based on standard procedures (i.e., without shoes but fully clothed). Height was measured to the nearest 0.1 cm using a stadiometer (Seca 206, Seca GmbH & Co., Hamburg, Germany) and weight was measured to the nearest 0.1 kg using a digital scale (Seca Bella 840, Seca GmbH & Co.). The body mass index (BMI) for each child was calculated using the formula: weight [kg]/height^2^[m]. BMI percentiles and BMI *z*-scores were calculated because BMI during childhood is age- and sex-specific. They are representative of current BMI (*z*-score) standards for Dutch children (Cole and Roede, 1999; Cole et al., 2000; Stichting Voedingscentrum Nederland, 2011).

### Analytical strategy

#### General tendency to imitate

In the first analysis, it was investigated whether imitation of food picking was likely to occur among children. Estimates were calculated to indicate whether the participants were more likely to imitate the confederate's food picking. The estimates were calculated by dividing the total duration of the social interaction (i.e., 10 min) into “sensitive” and “non-sensitive” periods. Within the 5 s-time window, the sensitive period was the 5-s interval which started when the confederate's finger had touched the test food (sensitive in terms of the likelihood of imitation). Since the confederate was signaled by the experimenter to pick a peanut each minute, there were 10 sensitive periods of 5s each^1^. The non-sensitive periods were all the remaining time periods (e.g., total time in seconds (=600) minus the sensitive periods (=50) equals 550). Next, the number of chocolate peanuts that were picked within those sensitive periods was divided by 50s (i.e., the sensitive period). The estimate for the non-imitated food picks represents how often participants picked a chocolate peanut in the non-sensitive periods divided by 550 (i.e., the non-sensitive period; outside the 5-s interval after the confederate picked a peanut). These two estimates were computed separately for each participant. To express whether participants had a general tendency to imitate, the sensitive and the non-sensitive estimates were tested with a paired sample *t*-test. A significantly higher sensitive than non-sensitive estimate indicates that participants are more likely to pick a peanut when cued by the confederates' food picking.

#### Likelihood of imitation of food picking over the duration of the social interaction

The second type of analysis aimed to test whether normal-weight and overweight children showed a different pattern in the likelihood of imitation during the social interaction in general. Therefore, the 10-min session was split into halves (referred to as the first 5-min and the second 5-min). The second 5-min was dummy coded as “1” vs. the first 5-min that was used as a reference category and coded as “0.” Further, overweight children were dummy coded “1” vs. normal-weight children which were used as a reference category and coded as “0.” A multilevel proportional hazard model (Cox regression) in a survival analysis framework was used to test whether the likelihood of imitation of the confederate's food picking depended on the duration of the social interaction (i.e., in the first 5-min or the second 5-min, which is used as a time varying predictor in the survival analysis) and the participants' weight status (which is used as a between-subjects predictor in the survival analysis). The total number of times the participant had picked chocolate peanuts was controlled for.

Hazard ratios (HRs) and confidence intervals (CIs) were presented as effect sizes. A hazard ratio is an expression of the hazard of events (i.e., imitation of food intake) occurring in one group (coded as “1”) as a ratio of the hazard of the events occurring in the reference group (coded as “0”). A hazard ratio above 1 indicates a higher hazard of events whereas a hazard ratio below 1 a reduced hazard of events. The significance level for all statistical tests was set at 5% (*P* < 0.05). Data were analyzed using SPSS 19.0 and MPLUS 5.1. (Muthén and Muthén, 2007). To correct for the skewed distribution in weight status, the Maximum Likelihood Robust estimator (MLR) was used in the Cox regression. MLR is robust to non-normality and non-independence of observations (Muthén and Muthén, 2007).

### Additional analysis

Additional analyses were performed to explore the likelihood of imitation of the confederate's food picking into more detail. Therefore, separate analyses (multilevel proportional hazard model (Cox regression) in a survival analysis framework) were conducted for the weight groups (normal-weight vs. overweight participants) and the duration of the social interaction (first 5-min vs. second 5-min of the experiment), controlling for the total number of times that participants picked chocolate peanuts.

## Results

### Descriptives

The means and standard deviations (SDs) for the variables age, sex, hunger, dietary restraint and total number of times participants picked chocolate peanuts are summarized in Table 1. No significant differences (*P* > 0.05) were found between the two weight groups. Table 2 presents participants' food picking for the first and second 5-min of the experimental session. The confederate followed instructions and picked food each minute throughout the session (*M* = 9.9, *SD* ± 0.7). A 2 × 2 repeated measure ANOVA with weight status as the between-subjects factor and time (first 5-min and second 5-min) as the within-subjects factor showed that overweight and normal-weight children did not significantly differ in the total number of times they picked test food during the 10-min social interaction [*F*_(1, 66)_ = 3.51, *P* = 0.07]. In addition, they did not significantly differ in the number of times they picked test food within the first or second 5-min (*F* < 1).

**Table 1:** **Means (*SD*) for variables age, sex, hunger, dietary restraint, and total number of picked chocolate-coated peanuts measured by weight status^1^**.

**Variables**	**Total (***n*** = **68**)**	**Normal-weight (***n*** = **49**)**	**Overweight (***n*** = **19**)**	***P*-value^**2**^**
Age (y)	8.56 ± 1.46	8.55 ± 1.61	8.58 ± 1.02	0.94
Boys/girls (*n*/*n*)	34/34	25/24	9/10	0.79
Hunger	3.71 ± 3.89	3.58 ± 3.83	4.04 ± 4.14	0.66
Dietary restraint	0.59 ± 0.45	0.53 ± 0.46	0.74 ± 0.39	0.09
Number of picked chocolate-peanuts	7.63 ± 6.39	6.75 ± 5.46	9.89 ± 8.07	0.07

1 All values are in means (±SDs).

2 P-values reflect the differences in total means between weight groups by one-factor ANOVA or Pearson's chi square test.

**Table 2:** **Food picking movements measured by the first and second 5-min of the social interaction^1^**.

	**Total (***n*** = **68**)**	**First 5-min**	**Second 5-min**
Food picking normal-weight participants (*n* = 49)	6.76 ± 5.46	3.39 ± (3.09)	3.33 ± (2.98)
Food picking overweight participants (*n* = 19)	9.89 ± 8.07	4.68 ± (4.27)	5.21 ± (3.97)
Food picking movements all participants	7.63 ± 6.39	3.75 ± (3.47)	3.85 ± (3.36)

1 Data are presented as means (±SDs).

### Main analysis

#### General tendency to imitate

The first analysis showed by means of a paired sample *t*-test that the participants were more likely to pick a chocolate peanut directly after the confederate had picked one than when the confederate had not reached for a peanut [*t*_(67)_ = 5.69, *P* < 0.0001]. This shows that children are likely to imitate and pick food when triggered by the confederates' food picking.

#### Likelihood of imitation of food picking

There was an interaction effect (*HR* = 2.6, *P* = 0.03, 95% *CI* = 1.09–6.20) between the duration of the social interaction and participants' weight status on the likelihood of imitation, controlled for the total number of times participants picked chocolate peanuts (*HR* = 1.09, *P* < 0.001, 95% *CI* = 1.05–1.12). The interaction effect indicates that there is a difference in the likelihood of imitation of the confederate's food picking between normal-weight and overweight participants over the time course of the social interaction. It was also tested whether sex, hunger, restrained eating or liking of the task had an effect on the main findings, but this was not the case (all *P*-values > 0.05).

### Additional analysis

To explore the interaction effect into more detail, separate analyses had to be conducted for the normal-weight vs. overweight participants and the first vs. second 5-min of the experiment, controlled for the total number of times participants picked chocolate peanuts. Table 3 shows the likelihood of imitation for the interaction effect and the separate analyses. The hazard ratios of the slope across time shows that overweight participants are almost twice more likely (*HR* = 1.88, *P* = 0.004, 95% *CI* = 1.23–2.86) to imitate in the second 5-min compared to the first 5-min whereas normal-weight participants have a smaller chance (*HR* = 0.51, *P* = 0.01, 95% *CI* = 0.30–0.86) to imitate in the second 5-min than in the first 5-min. Further, the mean difference in the likelihood of imitation between the normal-weight and overweight participants was only significant during the first 5-min in which overweight participants had a smaller chance (*HR* = 0.47, *P* = 0.02, 95% *CI* = 0.25–0.88) to imitate than normal-weight children.

**Table 3:** **Likelihood of imitation of normal-weight vs. overweight children controlled for total number of times participants picked food**.

	**Time frame of 5 s**	
	**HR**	**CI**
Weight status	0.46^*^	0.24–0.87
Duration social interaction	0.57^*^	0.35–0.94
Weight * duration social interaction	2.60^*^	1.09–6.20
Total number of food picks participant	1.09^***^	1.05–1.12
**SLOPE ACROSS DURATION SOCIAL INTERACTION^1^**		
Normal-weight children	0.51^**^	0.30–0.86
Total number of food picks participant	1.07^**^	1.02–1.11
Overweight children	1.88^**^	1.23–2.86
Total number of food picks participant	1.12^***^	1.07–1.17
**DIFFERENCE NORMAL- VS. OVERWEIGHT^1^**		
First 5-min	0.47^*^	0.25–0.88
Total number of food picks participant	1.07^***^	1.03–1.12
Second 5-min	1.19	0.59–2.41
Total number of food picks participant	1.09^***^	1.05–1.13

* P < 0.05,

** P < 0.01,

*** P < 0.001.

1 Overweight participants were almost twice (HR = 1.88) as likely to imitate in the second compared to the first 5-min, whereas normal-weight participants were 49% (HR = 0.51) less likely to imitate. Within the first 5-min, overweight participants were 53% less likely (HR = 0.47) to imitate compared to normal-weight participants.

## Discussion

The present study investigated imitation of palatable food intake among normal-weight and overweight children. The findings showed that children were more likely to eat after witnessing a peer reaching for snack food than without such a cue. In addition, children displayed different imitation responses based on their weight status. By the examination of the pattern of imitation, the findings showed that overweight children are more likely to imitate a peer's food intake in the second half of the social interaction than in the first half, whereas the opposite is true for normal-weight children. In addition, the likelihood of imitation in normal-weight children was significantly higher than in overweight children in the first half of the social interaction. These findings suggest that that children's imitation food picking movements may partly contribute to social modeling effects on palatable food intake.

The findings support the hypothesis that a peer reaching for food is likely to trigger children's snack intake. This is in line with findings on adolescents' imitation in eating (Hermans et al., 2012) and drinking (Koordeman et al., 2011). In a previous study on imitation effects on food intake, it was proposed that it is possible that people might automatically imitate the behavior of another person and by doing so, they might synchronize their eating behavior toward that of their eating companion (Hermans et al., 2012). Research supports the idea that observing a peer reaching for food might trigger an automatic response and guide one's own action to corresponding behavior (Lakin and Chartrand, 2003; Rizzolatti and Craighero, 2004; Knoblich and Sebanz, 2008; Chartrand et al., 2009). One potential mechanism that might explain these imitation responses is by means of the mirror-neuron system: ingestive mirror-neurons have been found to respond to observations of movements related to ingestive purposes, such as picking food, biting, or sucking (Rizzolatti and Craighero, 2004; Chartrand and van Baaren, 2009). Physical movements preceding the action (e.g., picking food) as well as the actual action itself (e.g., eating food) activate the mirror-neuron system (Rizzolatti and Craighero, 2004; Knoblich and Sebanz, 2008). Nevertheless, the authors acknowledge that the findings provide no information with regard to any underlying processes or mechanism(s). Therefore, it can only be speculated whether imitation responses are automatic. The current study also showed that children did not imitate the behavior of their peer all the time and that normal- and overweight children differed in their likelihood of imitation during the course of the interaction. These findings suggest that other (e.g., social) processes play a role in imitation responses as well.

Research has provided evidence to postulate that the differences in the likelihood of imitation between weight groups might be explained by affiliation purposes. Imitation of others is likely to be activated by a higher order goal to affiliate (Lakin and Chartrand, 2003; Chartrand et al., 2009), which might lead to an increased liking between persons (Chartrand and Bargh, 1999). Individuals failing in their affiliation goal, however, might be more likely to increase their efforts to ingratiate (Chartrand et al., 2009) In line with previous findings on imitation in normal-weight participants (Larsen et al., 2010; Hermans et al., 2012) a possible explanation for the decrease in likelihood of imitation in normal-weight children is that they perceived themselves to have achieved their affiliation goal. Following this tentative line of reasoning, overweight children might not have perceived social confidence from their peer by which they increased imitation (Pierce and Wardle, 1997). If so, one could assume that overweight children would have imitated the peer from the start of the social interaction. In contrast, overweight children followed a normal-weight peer's food picking even less than normal-weight children, which brings up the question whether they were preoccupied with ingratiation processes. As this study did not include such measurements, future research is needed to investigate this proposition. These studies could, for instance, pair children with either overweight or normal-weight confederates and could code both children's gestures, smiles, and social responses in order to gain more insight into differences in affiliative efforts among both weight groups.

An alternative explanation for our findings is that normal- and overweight children might show a difference in responsiveness or sensitivity to food cues (e.g., the presence of the food bowl). As the findings show that normal-weight and overweight children started off with a significantly different imitation pattern (while picking a similar number of times), overweight children might be more sensitive to the presence and sight of snack food than to the peer's food picking. The peer reaching for food might have functioned as an additional cue on top of the sight of the food that triggered overweight children to pick food and imitate later. Normal-weight children might have been triggered by the specific cue of a peer picking food at the start of the social interaction. Studies on food-related attentional biases have demonstrated that overweight individuals automatically direct their attention to food-related stimuli and to a greater extent than normal-weight individuals (Castellanos et al., 2009; Nijs et al., 2010; Yokum et al., 2011), however, findings have not been consistent with regard to whether this attention is maintained over time (Castellanos et al., 2009; Nijs et al., 2010) With regard to the present study, it might be that the attention to a peer reaching for food as an additional stimulus has decreased in normal-weight children but increased in overweight children. In addition, the findings might imply that normal-weight children became less responsive to the food picking cues of a peer during the social interaction, whereas overweight children became more responsive to such cues. Research has shown that normal-weight children are less sensitive to triggers of (over)eating than overweight children (Jansen et al., 2003) and have more response inhibition (Nederkoorn et al., 2006; Braet et al., 2007). Given that there is no research into increasing or diminishing response inhibition over time, the interpretation of the current findings warrants caution and further research is needed.

As the study was only the first in young people and exploratory in nature, a limitation of this study was that the findings provide no information with regard to which underlying processes or mechanism(s) were at play. In addition, the study does not include measures on affiliation. One could question the reliability of self-reported measures on how much participants want to affiliate with their co-eater assessed before, during, or after a social interaction. Future studies should take appropriate measures of affiliative bonding into account, for example, by coding gestures or smiles. Also, there were fewer overweight than normal-weight participants in the study sample. It was not possible at the time of data collection to realize similar sample sizes, which might have caused power issues in the current analysis. Replication in larger samples is required to draw more definite conclusions. As research revealed associations between adiposity level and social relationships with peers [see for review Puhl and Latner (2007)], it would be insightful to investigate whether imitation patterns look different, for example, within overweight (or obese) dyads vs. overweight/normal-weight dyads.

In conclusion, the influence of others on our food intake is a complex process in which different theoretical perspectives might overlap. As this study was only exploratory in nature, it is inconclusive why people imitate food intake and whether imitation responses are in fact direct or automatic. Nevertheless, this study provides preliminary evidence that young people follow the actions of peers when snacking together and this response occurs within a few seconds. Food picking by peers is likely to trigger children to eat snack food. Future research is warranted to test the value of those perspectives in the context of imitation of food intake. The findings might provide potential areas for the prevention of (over)consumption or support the intake of low energy-dense foods e.g., parents and schools could provide an environment which encourages children to snack healthy foods or limit unhealthy eating.

### Conflict of interest statement

The authors declare that the research was conducted in the absence of any commercial or financial relationships that could be construed as a potential conflict of interest.

## Footnotes

^1^The number of food picking cues given by the confederate was accounted for in the analyses. For example, when the confederate picked 9 times, there were 9 sensitive periods of 5s each, which equals a sensitive period of 45 and a non-sensitive period of 555 s.
